# Direct Detection of Lyme Borrelia: Recent Advancement and Use of Aptamer Technology

**DOI:** 10.3390/biomedicines11102818

**Published:** 2023-10-18

**Authors:** Nik Abdul Aziz Nik Kamarudin, Christina Injan Mawang, Mariana Ahamad

**Affiliations:** Acarology Unit, Infectious Disease Research Center, Institute for Medical Research, National Institutes of Health, Ministry of Health Malaysia, Setia Alam 40170, Malaysia; christinainjan@moh.gov.my (C.I.M.); marianaa@moh.gov.my (M.A.)

**Keywords:** Lyme disease, tick-borne disease, direct detection, aptamer, biosensor

## Abstract

*Borrelia burgdorferi* sensu lato (*B. burgdorferi* s.l.), which is predominantly spread by ticks, is the cause of Lyme disease (LD), also known as Lyme borreliosis, one of the zoonotic diseases affecting people. In recent years, LD has become more prevalent worldwide, even in countries with no prior records. Currently, Lyme Borrelia detection is achieved through nucleic acid amplification, antigen detection, microscopy, and in vitro culture. Nevertheless, these methods lack sensitivity in the early phase of the disease and, thus, are unable to confirm active infection. This review briefly discusses the existing direct detection methods of LD. Furthermore, this review also introduces the use of aptamer technology integrated with biosensor platforms to detect the Borrelia antigen. This aptamer technology could be explored using other biosensor platforms targeting whole Borrelia cells or specific molecules to enhance Borrelia detection in the future.

## 1. Introduction

Lyme disease (LD), or Lyme borreliosis, is caused by a bacterial infection from *Borrelia burgdorferi* sensu lato (*B. burgdorferi* s.l.). This vector-borne zoonotic disease is primarily spread by ticks from the genus *Ixodes*. The typical clinical manifestation of early infection is erythema migrans (EM) rash at the site, which occurs in >80% of patients in both Europe and the United States [1]. LD symptoms may range from asymptomatic to severe and develop into encephalitis and arthritis without prompt treatment. Other non-specific symptoms include fever, malaise, and myalgia; hence they are often misdiagnosed or left untreated. Currently, LD is prevalent in the United States and Europe [1], but diagnosing LD has become challenging as clinical manifestations vary between *B. burgdorferi* genospecies and disease progression differs between patients [2,3,4]. For example, rheumatological manifestations of LD are common in North America, while neurological manifestations are customary in Europe. In addition, acrodermatitis chronica atrophicans and lymphocytomas, usually caused by *Borrelia afzelii* or *Borrelia garinii*, are common in Europe and Asia but extremely rare in the United States [5,6]. In the United States, the new species *Borrelia mayonii* was recognized in the Upper Midwest region [7].

The global impact of LD has recently expanded to previously undetected regions and countries due to the booming international tourism in endemic nations, where the disease is under-reported by local healthcare practitioners [8]. For instance, LD cases have been documented among tourists in Brazil, Canada, Australia, and Japan [9,10,11,12,13]. The LD reservoir and vector hosts have migrated from their native habitats following the effect of climate change in the north, thus allowing *B. burgdorferi* to expand its territory northward by 250–500 km in the next 30 years [14]. Several studies have reported increasing LD incidences in various parts of Canada, Europe, and Asia, particularly China [14,15,16,17,18].

Malaysia has a tropical climate and abundant wildlife in local forests, making it an ideal breeding ground for ticks. *Ixodes granulatus*, a vector for the Borrelia pathogen, has been recorded in numerous areas throughout Peninsular Malaysia, but local LD occurrence is not well-reported [19,20,21,22,23,24]. In addition, *Borrelia yangtzensis* was isolated from *I. granulatus* ticks discovered on rodents in Selangor’s recreational forests (18.1%) and Sarawak’s oil palm plantations (72.2%) [21,25]. Clinical case reports from Japan and China have associated these new *Borrelia* genospecies with LD [26,27]. Moreover, unpublished data from the Acarology Unit, IMR, have reported that 47.4% of *Borrelia* spp. was isolated from ticks collected from four coastal locations in Selangor, and 73.3% of ticks from recreational forests in Malaysia carried Rickettsia and Borrelia. Therefore, the risk of Borrelia infection in the Malaysian population remains high despite the small number of confirmed LD infections, owing to underdiagnosis or a lack of sensitive detection tools.

Several seroprevalence studies in Malaysia reported that 153 serum samples from patients exhibiting various infectious disease symptoms showed that 16.3% of IgM and 3.3% of IgG were reactive to the complete antigen of *B. afzelii*. Meanwhile, 8.1% of serum samples among the aborigines in Peninsular Malaysia were reactive to *B. burgdorferi* s.l. genospecies [28]. These findings indicate the occurrence of co-infections and mixed infections in LD patients, such as leptospirosis, tick typhus, and melioidosis.

The LD diagnostic test comprises a two-tier serology test recommended by the Food and Drug Administration (FDA). Alternative detection assays with various sensitivities are employed in private laboratories as a confirmation tool for this disease. Currently, the direct detection test for LD is not easily accessible to the public. Therefore, this review discusses the existing direct detection methods of LD and the potential of aptamer technology integrated with a biosensor for Borrelia detection in various samples to enhance the sensitivity of detection tools.

## 2. Current Guidelines for LD Diagnostic Test

**Figure 1:**
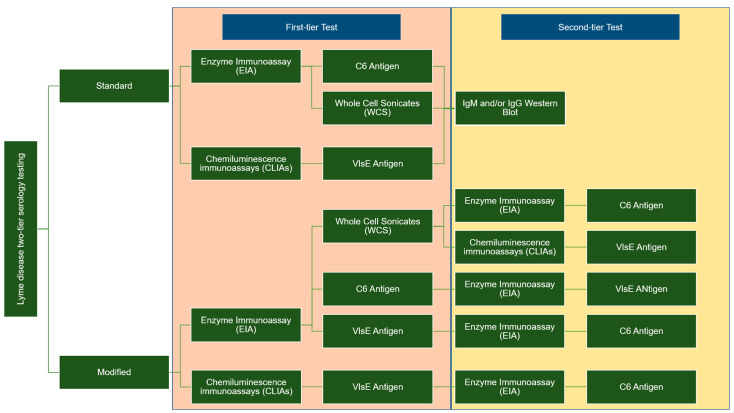
Differences between STTT and MTTT: Two-tier testing methods for LD diagnosis.

Two types of enzyme immunoassay, known as standard or modified, two-tiered serology testing (STTT/MTTT), are used to detect immunoglobulin (IgM or IgG). These methods differ in terms of the second confirmation assay; the STT uses western blotting, whereas the second enzyme immunoassay (EIA) is utilized for MTT when the sample is positive or ambiguous. Figure 1 summarises the difference between the two-tier STTT and MTTT testing [29,30,31,32].

The STTT sensitivity for LD is less than 50% in early localized infections, whereas the sensitivity can reach up to 100% in the late stages of the disease. The MTTT is consistent and more sensitive in early localized LD than STTT and demonstrated similar sensitivity in detecting late infections and specificities to STTT [33]. The two-tier testing is sufficient to rule in LD among patients who have tested positive in the early stages, but the tool has low predictive value to rule out LD, which necessitates retesting after 30 days [34]. Nevertheless, LD sensitivity in recovering patients treated at stage 1 remained low after 30 days. Resultantly, the diagnosis and treatment of early localized LD solely depend on the clinical symptoms of individuals with a history of exposure to black-legged ticks.

Early LD can be difficult to diagnose, as some individuals with localized *B. burgdorferi* infections do not have an EM rash and may exhibit symptoms similar to those of other diseases. As both STTT and MTTT cannot distinguish between active and past infection, *B. burgdorferi* antibodies can persist for months to years after the initial infection [35]. Despite the higher sensitivity than STTT, MTTT sensitivity is still <90%. Thus, patients with early localized LD should be treated based on their clinical presentation rather than serologic data [33].

The main pitfall of this assay is that it requires a complex laboratory infrastructure, producing inter- and intra-laboratory result variability, a long turnaround time, and a high cost for the immunoblot assay [36]. Eventually, this assay will only be based on yes-or-no results for routine cases of suspected Lyme disease. Other limitations include a high background rate of seropositivity in the endemic area, antibody cross-reactivity with other related bacterial infections, and false-positive results due to other medical conditions [37].

## 3. Direct Detection Method of Borrelia

### 3.1. Nucleic Acid Amplification

Most commercial kits are developed to target and conserve the genomic sequences of Borrelia species using two-step PCR (nested PCR). Borrelia species can be directly detected in patients’ samples, such as synovial fluids, cerebrospinal fluid (CSF), and blood, using polymerase chain reaction (PCR). The PCR assay targets several genes located on the genome sequence or linear plasmid of Borrelia species, such as flagellin B (*flab*), outer surface protein A (*OspA*), *OspC rpoB*, and 16S rDNA [38,39,40]. These target genes are also useful for phylogenetic analysis of Borrelia from ticks. Nevertheless, this method demonstrates various sensitivity ranges (4–100%) with 93–100% specificity [41]. Furthermore, the sensitivity ranges between 5 and 50% for EM skin biopsies compared to the CSF samples [42,43]. The low DNA recovery in patients’ samples, particularly for younger patients with limited samples, reflects the unsatisfactory sensitivity of the assay.

Several PCR protocols have been established to enhance the sensitivity of the detection method in combination with the enrichment step. For instance, the loop-mediated isothermal amplification (LAMP) assay targets the 16S rRNA and has higher sensitivity (0.2 to 0.02 pg of DNA) in detecting *B. burgdorferi* s.l. isolated from field-collected ticks compared to conventional and nested PCR [44]. In another study, the LAMP assay targeted the flagellin (*fla*) gene to detect as few as 20 copies of DNA per reaction and cross-react with 11 related bacteria [45]. Thus, the success rate of the LAMP assay is equivalent to nested PCR. Furthermore, the high amplification efficiency of the LAMP assay is due to the continuous amplification under isothermal conditions, yielding magnesium pyrophosphate as a by-product. The white-coloured precipitation is easily observed by the naked eye or by real-time turbidity monitoring using the conventional photometer [46].

Recombinase polymerase amplification (RPA) is another example of an isothermal amplification method that utilizes the recombinase protein to unwind the double-stranded DNA molecules and the strand-displacing activity to amplify DNA targets. This process takes 20–30 min from 37 °C to 42 °C [47]. The detection limit of the RPA assay targeting the *recA* gene is five copies, while the sensitivity is 50 femtograms of *B. burgdorferi* genomic DNA [48].

Even though the PCR sensitivity is high, this assay cannot differentiate between live and dead bacteria within the samples. The positive PCR result is unable to distinguish live bacteria in patients with persistent arthritis after antibiotic therapy [49]. In addition, PCR sensitivity is also highly variable depending on the type of starting materials, DNA extraction methods, target gene location, and PCR amplification method [50,51,52,53,54]. Thus, further evaluation of the PCR assay is still required to improve the assay sensitivity, and the method must be standardized to provide unambiguous diagnostic results.

### 3.2. Direct Antigen Detection

Borrelia antigen detection is a dependable, quick, accurate, and sensitive method for early LD diagnosis and improved patient care before acquiring disseminated LD. These assays are performed using Borrelia polyclonal antibodies, with the shed surface antigens detectable in the urine, blood, and various organs of infected hosts [55]. Nonetheless, the assay sensitivity, specificity, and accuracy in detecting several antigenic moieties (31 kDa, 34 kDa, 39 kDa, and 93 kDa) are reportedly low in invalidated clinical samples [56]. The assay can be enhanced by performing high-speed centrifugation to separate Borrelia membrane proteins from the rest of the serum proteins. *B. burgdorferi* cells that have been broken will release membrane vesicles containing membrane proteins into the blood. Precisely, high-speed centrifugation contributes to a low protein detection limit at approximately 4.0 fmol of ospA/mg of serum protein [57].

Hydrogel microparticles are widely used in biomedical research, such as being incorporated with chemical bait to mediate small target molecule sequestration and entrapment. This method helps concentrate the target molecules in the solution and eliminate other molecules through sieving. For instance, Douglas et al. reported that Acid Black 48 dye effectively acts as bait to concentrate the *B. burgdorferi* OspA and OspB proteins at a limit of detection (LOD) of 700 pg/mL in 10 mL of urine [58]. In another study, Remazol Brilliant Blue dye was used as bait for the Nanotrap technology. The Nanotrap particles were coupled with a Borrelia monoclonal antibody that targeted epitope OspA 236–239, with a detection rate as low as 1.7 pg/mL of Borrelia antigen. These studies supported the shedding of urinary OspA protein, which strongly correlates with the clinical diagnosis of the early and active stage of LD [59].

An earlier study found ten highly conserved amino acids in OspC that are significantly immunodominant and potential immunogens for anti-OspC antibody production [60]. In addition, this peptide contains a common sequence (PVVAESPKKP) found in most pathogenic Borrelia [60,61]. A solid-phase immobilized epitope immunoassay (SPIE-IA) of OspC protein was recently developed [62], despite the high variability of this protein within and between the Borrelia species. The developed SPIE-IA recorded a LOD as low as 17 pg/mL for OspC from infected animal blood samples. Moreover, the study detected a mean value of 10 ng/mL OspC in the plasma of infected mice at seven days post-infection [62].

### 3.3. Biosensor

**Figure 2:**
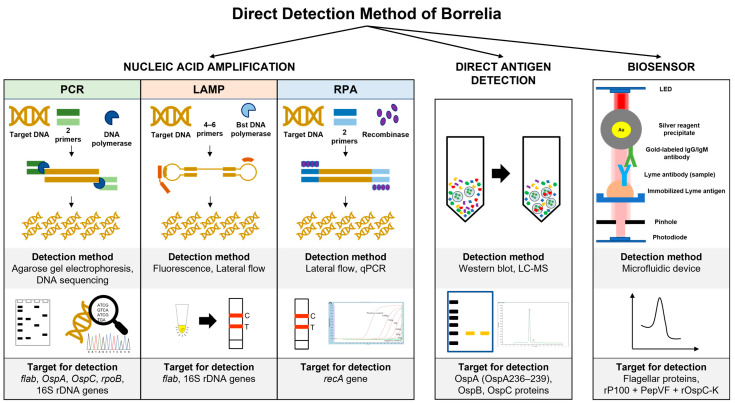
Direct detection method of Borrelia using isolated genetic material and soluble antigen in various samples. The biosensor detection diagram was modified from [54], which is open access under the Creative Commons Attribution 4.0 International license.

Several single multiplexed assays have been developed to replace the second tier of serology assays. For example, the mChip-Ld assay targeted three Borrelia antigens (VlsE, PepVF, and OspC) and showed a sensitivity range between 80% and 100% in sample patients with early Lyme disease and Lyme arthritis, respectively [36]. Several biosensor applications have been developed to detect Borrelia antigen, including field-effect transistors (FETs) and microfluidics [63,64]. The attachment of *B. Burgdorferi* flagellar antibodies (p41) to nanotubes effectively detected antigens in buffer (1 ng/mL) using atomic force microscopy [63,64]. The assay utilized the turnoff voltage measurement following antigen exposure, which involves shifting the threshold voltage towards the more negative region. Furthermore, the device is highly selective for the target antigen on the flagellar protein. In a different study, the PPO triplex test (rP100 + PepVF + rOspC-K)-based microfluidic diagnostic device (mChip-Ld) was developed for antibody detection during early and late LD stages. The assay recorded 84% sensitivity and 92% specificity, comparable to the lab-based C6 peptide ELISA [64]. Figure 2 summarises the method used for direct detection of genetic material and soluble antigens of Borrelia.

## 4. Aptamer for Borrelia Antigen Detection

The aptamer is a single-stranded nucleic acid (RNA or DNA) that can bind with various target molecules, including surface proteins, viruses, bacteria, nanoparticles, and small analytes. The aptamer can bind to target molecules with low nanomolar and picomolar affinity and distinguish the target molecule from other similar molecular structures or functional groups [65]. Furthermore, the aptamer can form a compatible tertiary structure that fits into the binding pocket of the target molecules through hydrogen bonds and hydrophobic interaction.

The aptamer can be isolated in vitro via the Systematic Evolution of Ligands by Exponential Enrichment (SELEX). This method allows large oligonucleotide libraries to undergo several enrichment cycles of bound aptamer, which involves binding, washing, and eluting, to produce the best aptamer with high affinity and specificity towards the target molecules.

The use of aptamers as diagnostic tools has been widely explored in managing infectious diseases. Xenobiotic nucleic acid (XNA) is a new aptamer variety that has been extensively studied due to its intrinsic resistance to cellular nucleases [66]. The direct evolution of specialized XNA polymerase has promoted the enzymatic production and enrichment of XNA libraries to synthesize XNA sequences from DNA templates before being transcribed into the DNA [67,68,69]. Aptamer detection has been applied to various targets of infectious diseases, such as SARS-CoV, SARS-CoV-2, *Mycobacterium tuberculosis* (*M. tb*), and melioidosis [70,71]. Several aptamers targeting tick-borne diseases have been developed, such as surface protein E of the tick-borne encephalitis virus (TBEV) and the *B. burgdorferi* outer surface protein (ospA, ospC, and BmpA) associated with LD [72].

There are several advantages to the application of aptamers as a diagnostic tool, such as ease of chemical synthesis and modification, low manufacturing costs, thermal stability, and minimum batch-to-batch variation for mass production. Despite the susceptibility to nuclease degradation, the sugar ring, nucleotide bases, and phosphodiester bond modification can improve aptamer structural stability in the nuclease environment and enhance the binding affinity to the target molecules [73]. The aptamer can be modified at the 3′ or 5′ end with functional groups (thiol, amine, carboxyl) or biomolecules (biotin, fluorophore) for capturing and detection purposes [reviewed by [74,75,76].

Regarding the detection of Borrelia antigen, the aptamer can be used to detect the presence of soluble proteins released by the bacteria or whole bacteria in the collected samples. Until now, various samples have been tested to detect the presence of Borrelia directly or indirectly from blood serum, cerebrospinal fluid (CSF), urine, skin biopsy, and synovial fluid [77,78,79]. This bacteria’s antigen is often shed into the body fluids, which can later be detected using the aptamer. Interestingly, aptamers can function as reporters and capture agents for direct detection. Several surface proteins of Borrelia have potentially been used, such as OspA, OspC, flagellin, BBK32, PepVF, VlsE, BmpA, DbpA, and DbpB [64,80,81].

## 5. Current Aptamer Development for Borrelia Antigen Detection

Only one study has used aptamer technology to directly detect Borrelia antigen using surface-enhanced Raman spectroscopy (SERS) [82]. SERS is an ultrasensitive method for single-molecule detection, which is a powerful tool for biosensing applications in various fields. Furthermore, SERS peaks have narrow bandwidths and a characteristic molecular “fingerprint” compared to other techniques; thus, this method is ideal for multiplex detection. The unique surface plasmon resonance of metallic nanoparticles is utilized in this technology, which operates as signal-amplifying substrates and eliminates the need for pathogen culture [83]. Surface-bound, highly affine capture biomolecules provide a layer of specificity to these particles, allowing whole-cell pathogen fingerprinting.

The design of highly sensitive Raman aptasensors requires rigorous control of the three-dimensional assembled configuration to maximize SERS enhancement, particularly the local amplification of the electromagnetic (EM) field. Moreover, SERS aptasensors were developed by combining SERS probes with highly sensitive and selective aptamers for recognizing target molecules [84]. In a recent study, SERS accurately identified 91% of serum samples from Lyme patients and 96% of serum samples from symptomatic controls with a LOD of 1 × 10^−4^ ng/mL, more than four orders of magnitude lower than serum samples from early LD patients [82].

This assay demonstrated a 50% improvement in sensitivity without significantly reducing specificity, unlike the existing Lyme diagnostic assay. In other studies, SERS identified bacteria from complex solutions with a high capture efficiency for *S. aureus* (88.89%) and *E. coli* (74.96%) within 15 min [85,86]. The latest applications of the other SERS-based aptasensor include the detection of the SARS-CoV-2 virus, *Salmonella* Typhimurium, H3N2 virus, *Vibrio parahaemolyticus*, *Staphylococcus aureus*, and influenza A [87,88,89,90,91,92]. Table 1 compares the available detection methods for Lyme disease and their sensitivity.

## 6. Future Direction of the Use of LD Aptasensor

Integrating the aptamer technology with the available sensor platform might improve the sensitivity and specificity for direct detection of Borrelia antigen. As discussed previously, several biosensor platforms have been developed for direct antigen detection for LD using the antibody, such as microfluidics, lateral flow assays (LFAs), vertical flow assays (VFAs), surface plasmon resonance, and biochips [93]. This antibody-based biosensor can be replaced by an aptamer that might give better performance. The aptamer can easily be modified to conjugate with any nanoparticle, such as gold, graphene oxide, and fluorescence dye, which can later be used as capturing or reporter agents [94,95,96]. Nowadays, the label-free aptamer is used with quencher molecules such as graphene oxide that can turn “on” the sensor when the aptamer binds to a specific target with a detection limit of 1.26 pg/mL [97]. The yes-or-no test might suit LD diagnosis when a low amount of antigen is present in the samples and eliminates the need for an enrichment step. Other than that, aptasensor development for direct detection of Borrelia might be able to differentiate between live and dead bacteria. This could be performed using the whole-bacteria aptamer SELEX (Systematic Evolution of Ligands by Exponential Enrichment) using live Borrelia and heat-killed Borrelia. This whole-cell SELEX was successfully developed to detect live *Salmonella* Typhimurium and *Lactobacillus acidophilus* down to 600 CFU mL^−1^ and 106 CFU mL^−1^, respectively [98,99]. This aptasensor might give a superior result compared to PCR, which is unable to differentiate dead bacteria from viable bacteria. The use of aptamers for direct detection of Borrelia integrated with available or new sensor technology might change the way LD is diagnosed in the near future. 

## 7. Conclusions

The direct detection of Borrelia antigen or whole bacterial cells is a promising tool in the early identification of LD for improved patient management. The aptamer is an advanced technology with the potential for Borrelia antigen detection. Notably, combining the latest technology with the aptamer could enhance test sensitivity and detection limits and reduce the time required to complete the assay. Furthermore, the test can function alone or complement the conventional serological test practiced in most laboratories. In summary, a fast and convenient assay may facilitate the diagnosis of the fever-like symptom possibly caused by Lyme Borrelia infection.

## Figures and Tables

**Table 1:** The comparison of the type of detection, assay sensitivity, and limit of detection for Lyme disease diagnostic and Borrelia direct detection.

Detection Methods	Target Molecules	Sensitivity	LOD	Reference
Nested PCR	*p66*, 16S rRNA gene, *fla* gene, 23S rRNA gene, 5S rRNA-23S rRNA gene spacer, *recA* gene, *OspA* gene	4–100%	ND	[41,42,43]
LAMP	16S rRNA	32.7%	0.2 to 0.02 pg	[44]
	*fla* gene	37.5%	20 copies	[45]
RPA	*recA* gene	90%	50 femtograms	[48]
Hydrogel microparticles	*B. burgdorferi* OspA and OspB	ND	700 pg/mL	[58]
Nanotrap technology	OspA 236–239	87.5%	1.7 pg/mL	[59]
SPIE-IA	OspC	ND	10 ng/mL–17 pg/mL	[62]
mChip-Ld	VlsE, PepVF, OspC	80% to 100%	ND	[36]
FETs	p41	Unavailable	1 ng/mL	[63]
Microfluidics	rP100, PepVF, rOspC-K	84%	ND	[64]
SERS (aptasensor)	OspA	91–96%	1 × 10^4^ ng/mL	[83]
